# Coralline algal physiology is more adversely affected by elevated temperature than reduced pH

**DOI:** 10.1038/srep19030

**Published:** 2016-01-07

**Authors:** Román Manuel Vásquez-Elizondo, Susana Enríquez

**Affiliations:** 1 Unidad Académica de Sistemas Arrecifales Puerto Morelos (Reef System Academic Unit), Instituto de Ciencias del Mar y Limnología, Universidad Nacional Autónoma de México (ICML-UNAM), Apartado postal 1152, Cancún, Q. Roo 77500, Mexico

## Abstract

In this study we analyzed the physiological responses of coralline algae to ocean acidification (OA) and global warming, by exposing algal thalli of three species with contrasting photobiology and growth-form to reduced pH and elevated temperature. The analysis aimed to discern between direct and combined effects, while elucidating the role of light and photosynthesis inhibition in this response. We demonstrate the high sensitivity of coralline algae to photodamage under elevated temperature and its severe consequences on thallus photosynthesis and calcification rates. Moderate levels of light-stress, however, were maintained under reduced pH, resulting in no impact on algal photosynthesis, although moderate adverse effects on calcification rates were still observed. Accordingly, our results support the conclusion that global warming is a stronger threat to algal performance than OA, in particular in highly illuminated habitats such as coral reefs. We provide in this study a quantitative physiological model for the estimation of the impact of thermal-stress on coralline carbonate production, useful to foresee the impact of global warming on coralline contribution to reef carbon budgets, reef cementation, coral recruitment and the maintenance of reef biodiversity. This model, however, cannot yet account for the moderate physiological impact of low pH on coralline calcification.

Global warming and ocean acidification (OA) are two global threats that affect many different aspects related to the maintenance of biological communities, ecosystem services and human population stability. Therefore, they are major concerns in marine research, particularly with respect to calcifying organisms^1^,^2^,^3^. OA has received significant scientific attention relative to global warming, due to the increasing number of studies that have concluded that decreases in the calcium carbonate saturation state of seawater (CaCO_3_, Ω) have significant impact on calcifying organisms^3^,^4^,^5^,^6^. Meta-analyses initially questioned the significance of OA as a major threat to marine biodiversity^7^ but have recently documented an enhanced sensitivity of marine organisms to OA under elevated seawater temperature highlighting different adverse effects, such as declines in survival, calcification rates, growth, and abundance, in a broad number of organisms^6^.

Coralline red algae (Rhodophyta), key calcifying primary producers present in most coastal ecosystems^8^, are considered one of the most sensitive organisms to OA^9^. However, the sensitivity of coralline algae to increasing temperature is still unclear, as experimental analyses have documented opposite results^10^,^11^. Coralline algae precipitate high Mg-calcite^12^,^13^, the most soluble form of CaCO_3_, on their cell walls^12^ to form external coralline carbonate skeletons that represent more than 80% of the dry mass of the thallus^14^. These external skeletons provide structural support and protection but increase the vulnerability of the thalli to reduced pH^9^,^13^, in spite of skeletons are still protected from surrounding environment by an epithelium^15^. Coralline algae are important contributors to coral reef carbon budgets^16^,^17^,^18^,^19^ and play a key role in reef framework development^20^. Erect morphologies, such as articulated algae and rhodoliths, also provide habitats for a large diversity of organisms^21^,^22^,^23^, and crustose species (CCA) serve as a substrate for the settlement of coral larvae^20^,^24^. Given the ecological significance of coralline algae in many coastal ecosystems, the adverse impact of global change on this group will compromise a large variety of ecosystem processes^2^,^9^,^25^.

Investigations of coralline responses to OA have yielded contradictory results. Some studies have reported that OA decreases calcification^4^,^26^,^27^ and rates of photosynthesis^4^,^11^,^27^ and growth^28^,^29^ while others have not observed a clear effect of OA on algal metabolism^10^,^30^,^31^,^32^. Analyses of the effects of thermal stress have also produced results ranging from no effect^10^,^11^,^30^ to significant decreases in photosynthesis and calcification^4^. Several authors have also concluded that thermal stress exacerbates the adverse effect of OA on algal calcification^11^,^30^. Moreover, thermal stress triggers deleterious effects on coralline performance, such as a reduction of larval metamorphoses capacity, the induction of bleaching and tissue mortality for different CCA species^30^,^33^. These contradictory findings have been interpreted as resulting of a significant species-specific component of coralline sensitivity to global change.

Light stress and the inhibition of photosynthesis are fundamental in the analysis of the dysfunctional coralline response because there is a linear association between photosynthesis and calcification^12^,^34^. Unfortunately, these factors have received limited attention in the experimental analysis of coralline responses to global change, but recent studies of the effect of elevated temperature and *p*CO_2_ have provided new evidence of the dependence of coralline calcification on light and photosynthetic activity. These studies have concluded that irradiance is the only parameter that significantly impacts CCA calcification in *Hydrolithon reinboldii*^10^ and that calcification in the CCA *Lithophyllum cabiochae* is linearly related to photosynthesis^11^. Algal calcification is also related to thallus respiration because the former is energetically dependent on both photosynthesis^18^,^19^ and mitochondrial respiration.

The aim of this study was to contribute to the understanding of the physiological responses of coralline algae to reduced pH and elevated temperature by analyzing the direct and combined effects of OA (pH of 7.9) and thermal stress (+2 °C above the local maximum monthly mean [MMM] of 30 °C^35^) on thallus physiology. We focused the attention on the analysis of the role of light-stress in this response. Therefore, we selected the experimental light fields according to the particular photobiology of each species, and monitored the diurnal variation of solar irradiance and the impact caused on the maximum photochemical efficiency of photosystem II (F_v_/F_m_). We selected three species belonging to the three main coralline growth forms, which represent different ecological and/or evolutionary solutions with potential contrasting morpho-functional abilities to respond to environmental changes: the rhodolith *Neogoniolithon sp.;* a crustose coralline (CCA), *Lithothamnion sp.*; and an articulated alga, *Amphiroa tribulus* (Fig. 1). Specifically, we tested the direct and combined effects of both of these global stressors on thallus photosynthesis, respiration and calcification rates by focusing on their metabolic coupling and on the role of light stress and photosynthesis inhibition in the disruption of this coupling. Prior to this analysis, we characterized the physiology of the experimental organisms by determining: (1) the photosynthetic light response (P vs. E curve) for each species; (2) the species-specific physiological coupling among the photosynthesis, respiration and calcification rates; and (3) the short-term response of each metabolic rate to temperature (Q_10_).

## Results

### Photobiology and metabolic coupling among photosynthesis, respiration and calcification

In their optimal physiological condition in summer (30 °C), rhodoliths exhibited the highest metabolic rates, except at subsaturating light conditions, at which the lowest rates of photosynthesis were observed (lower photosynthetic efficiency, α, Fig. 2a; Table S1). By contrast, the CCA exhibited the lowest metabolic rates on an area basis (Figs 2 and 3). The photosynthetic efficiency of the CCA was as high as that of the articulated alga, which exhibited intermediate photosynthesis values (Fig. 2a). At subsaturating photosynthesis, we observed decalcification (negative values for calcification, G, Fig. 2) in the three corallines where the rhodolith had the highest decalcification rates. Also, we observed saturation of coralline calcification before saturation of photosynthesis, particularly in the articulated alga (Fig. 2b). This observation implies a non-linear association of coralline calcification (G) and gross photosynthesis (P) (Fig. 2b). A sigmoidal function provided a quantitative description of this coupling and an estimation of (i) maximum coralline calcification (G_max_, μmol O_2_ m^−2^ h^−1^; Fig. 2b) as the amplitude, (ii) *light enhanced calcification* (-r_G:P_; mol CaCO_3_ produced per mol O_2_ evolved; see Table 1) as the coralline calcification enhancement rate under increasing illumination and photosynthesis, and (iii) the magnitude of decalcification activity under subsaturating light. The sigmoidal function also highlighted a photosynthesis threshold for coralline calcification, referred here to as the *minimum photosynthesis requirement* (MPR, R^2^ > 0.80, Fig. 2b, Table 1), above which coralline calcification was positive and enhanced by increasing photosynthesis until the maximum rate was reached (G_max_, Fig. 2b). MPR was lower for CCA and the articulated alga, *A. tribulus* (Table 1), while the rhodolith *Neogoniolithon* sp. required a three-fold greater photosynthetic rate to offset thallus decalcification (Table 1). In contrast to this non-linear coupling, post-illumination respiration and coralline calcification exhibited a linear relationship (R^2^ ≥ 0.95, P < 0.01; Fig. 2c). The slope was similar for the CCA and the rhodoliths (0.573 ± 0.028; *F*(1,6) = 0.84, P = 0.39, Fig. 2c) and smaller for the articulated alga (0.363 ± 0.045, *F*(1,14) = 10.2, P = 0.009, Fig. 2c).

### Scaling quotient of temperature (Q_10_)

Short-term incubations evidenced that temperature, as expected, significantly enhanced all metabolic rates along an optimal physiological range (Fig. 3), but significant differences in metabolic rates were observed among species (Table 1 and Tables S2 and S3). Post-illumination respiration (R_L_) and thallus calcification (G_max_) increased linearly with temperature from 24 °C to 32 °C for the rhodolith and the articulated alga, whereas the CCA exhibited a decline above 30 °C (Fig. 3b,c). A similar decline in thallus photosynthesis (P_max_) was observed above 30 °C for the three species (Fig. 3b,c). At 30 °C, which represented the seawater conditions at the moment of sampling (August 2013) and the maximum average summer temperature for this reef lagoon, P_max_ for the three coralline species and all metabolic rates for the CCA reached a maximum (Fig. 3). The lowest values were measured at 24 °C (Fig. 3). The ratio between P_max_ and R_L_ (P:R) changed moderately with increasing temperature, particularly for the CCA (Fig. 3d), despite the significant changes in the photosynthesis and respiration rates. The articulated alga, *A. tribulus*, which exhibited larger reductions in P_max_ relative to R_L_ with increasing temperature, exhibited the greatest adverse effect of temperature on thallus P:R (Fig. 3d). A slight but non-significant reduction in thallus P:R was also observed under elevated temperatures in the rhodolith (Fig. 3d). The value of the Q_10_ factor ranged from 2 to 3 and differed between morphs and metabolic rates (Table 1 and Tables S2 and S3). P_max_ exhibited the highest Q_10_ in the rhodoliths and the lowest in CCA, whereas G_max_ displayed the opposite pattern, with the highest temperature enhancements in the CCA and the lowest in the rhodolith (Table 1; Table S1). By contrast, the effect of temperature on R_L_ was highest for the articulated alga and lowest for the CCA (Table 1 and Table S2).

### Variations in light exposure and water chemistry during the experiment

Organisms were exposed to diurnal variations in solar radiation under three different light treatments (see Materials and methods). Solar radiation exhibited minor changes during the 14 experimental days (August 18–31, 2013) with the exception of day 5, when we recorded a 50% reduction in light exposure due to dense cloud cover (Fig. S1). The average values at the surface exhibited maximum irradiance peaks of ~1700 μmol quanta m^−2^ s^−1^ at noon (Fig. S1) and an integrated daily light exposure of 35.2 ± 7.4 mol quanta m^−2^ day^−1^ (Fig. S1). We selected a specific experimental light regime for each coralline to induce similar, moderate light pressure (light stress) during the experimental treatments by adjusting each experimental light regime to the corresponding photobiology of the particular species. Rhodoliths, which were collected at a depth of 3 m within the seagrass bed, displayed a photoacclimatory response to higher light conditions compared to articulated alga and the CCA, with a higher P_max_ and Ek and lower photosynthetic efficiency, α (see P vs. E curves, Fig. 2a). The CCA and the articulated alga were collected at depths of 5–6 m within the coral community and were thus acclimated to lower light conditions. However, in a previous experiment in which the organisms were exposed to the reef light conditions at a depth of 5 m (37% of surface irradiance -%Es- and Kd = 0.198 m^−1^), we observed that the CCA bleached and exhibited 100% thallus mortality immediately after the exposure of one day to direct light. The CCA selected for this comparison was a species belonging to the genus *Lithothamnion* sp., which was collected underneath coral colonies of *Orbicella faveolata* and *O. annularis* and was thus exposed to much lower light conditions than the articulated alga (30–37% of Es at 5–6 m depth). Its shade-adapted response was reflected in the low P_max_ and Ek values and in the high photosynthetic efficiency, α (see P vs. E curve, Fig. 2a). The previous characterization of the P vs. E curves enabled the selection of the optimum experimental light regime for each species. We confirmed that a similar and moderate light pressure was induced on the three species during the experiment, as the variation in F_v_/F_m_ in the control organisms was minimal (see Fig. 4). Reductions in F_v_/F_m_ reflect the accumulation of photoinactivated photosystem II (PSII) due to photodamage, whereas F_v_/F_m_ recovery is indicative of positive PSII repair. The final experimental light regimes (diurnal light exposure) averaged 16.4 ± 0.91, 12.3 ± 0.68 and 0.35 ± 0.01 mol quanta m^−2^ d^−1^, respectively, for rhodoliths, articulated alga and CCA (see Fig. 4). The variations in water temperature, pH and carbonate system parameters among the treatments are illustrated in Table S4 in the Supporting Information.

### Chlorophyll a fluorescence

The variation in F_v_/F_m_ during the experiment was similar for the control organisms of the three species (Fig. 4), confirming that the different selected light regimes exerted similar pressure on the photosynthetic apparatus of each species. We also observed an initial decline in F_v_/F_m_ until day 3 that was of similar magnitude among species (~15%, Fig. 4). The initial F_v_/F_m_ decline was followed by a partial or complete F_v_/F_m_ recovery by day 5–6, when the light exposure minima were recorded (Fig. 4). These observations indicate i) the close dependence of F_v_/F_m_ on the diurnal variation of solar radiation, ii) the similar and moderate light stress induced by each experimental light treatment on the corresponding species, and iii) the ability of the control organisms of the three species to photoacclimate and cope with the experimental light stress. The control organisms did not exhibit significant changes in F_v_/F_m_ at the end of the experiment (Fig. 4), but significant variations in thallus pigmentation (Fig. S2, Table S5) and F_v_/F_m_ (Fig. 4, Table S6) were observed in the thermally stressed organisms. The largest accumulation of PSII-photodamage was observed in the articulated alga, *A. tribulus*, and the CCA, whereas the lowest accumulation occurred in the rhodolith (Fig. 4). We quantified a 45%, 30% and 20% reduction in the final F_v_/F_m_ values (experimental day 10) compared to the controls in the articulated alga, CCA and rhodolith, respectively.

### Physiological determinations

The metabolic rates of the control organisms remained unchanged during the experiment, which is indicative of a minimally adverse tank effect on algal physiology (Tables S7 and S8). However, comparison revealed significant differences among treatments and growth-morphs. Elevated temperatures induced large declines in P_max_ and R_L_ in all of the corallines (Two-way ANOVA, P < 0.05, Table 2 Fig. 5). Rhodoliths exhibited a 40% reduction relative to the control (Tukey HSD, P < 0.05), while the largest photosynthetic and respiratory losses (60–75%) were observed in the articulated alga and the CCA (Fig. 5a,b). No effect of low pH on thallus photosynthesis and respiration rate was observed, but we observed significant declines in G_max_ compared to the control rates of one half in the rhodolith and CCA and two thirds in the articulated alga (Tukey HSD, P < 0.05; Fig. 5c). Thermal stress caused similar reductions of thallus calcification in the rhodoliths (50% of the control), full inhibition of calcification in the CCA, and significant decalcification activity in the articulated alga *A. tribulus* (Fig. 5c). The combined effect of thermal stress and low pH resulted in reductions of P_max_ and R_L_ in the three species similar to those observed in the thermal stress treatment alone (Tukey HSD, P < 0.05). However, calcification rates (G_max_) revealed an additive effect in the rhodoliths and CCA and a significant interaction (synergistic effect) in the articulated alga *A. tribulus* (two-way ANOVA, P < 0.05, Table 3). Finally, minimal variation in the photosynthesis/respiration ratio (P:R) of the three species was observed among treatments, and a nearly constant value of ~2.5–3 mol O_2_ produced per mol O_2_ respired was observed (Fig. 5d)

## Discussion

The results of this experimental study showed significant adverse effects of elevated temperature and low pH on coralline algal physiology and wide differences in their physiological impact among the three species examined. Elevated temperature caused a progressive accumulation of photodamage, which was expressed as a continuous reduction of F_v_/F_m_ along the experiment in the three species. No accumulation of photodamage was observed in the control treatments (Fig. 4), indicative that the experimental light conditions were optimal and the three species were well photoacclimated to their respective light conditions. After exposure to elevated temperature (32 °C), we observed significant losses in algal photosynthesis and calcification in addition to the enhancement of photodamage, which were particularly pronounced for the articulated alga *Amphiroa tribulus* (Fig. 5). These findings indicate that light-induced photodamage was exacerbated under elevated temperature in the three coralline algae in spite of their contrasting photobiology, photoacclimatory condition and growth-form. This finding highlights the central role of light stress in determining the severity of the impact of thermal stress on coralline performance, consistent with previous results documented for CCAs^33^. It also reveals a similar response for coralline algae to that already documented for scleractinian corals^36^,^37^.

The limited attention paid to light stress in the analysis of the response of coralline algae to elevated temperature and low pH can explain the contrasting results and conclusions previously documented^4^,^10^,^11^. For example, the lack of impact of thermal stress on algal photosynthesis documented for the deep-growing CCA *L. cabiochae* by Martin, Cohu^11^, could have resulted from the very low light levels used in this study to simulate the natural light conditions of this species, insufficient to induce light stress on this species. In contrast, the strong decline in net photosynthesis observed by Anthony, Kline^4^ for the CCA *Porolithon onkodes* was likely due to the excessive experimental illumination applied in that study, which led to the induction of severe light stress on the experimental organisms. Anthony, Kline 4, however, attributed to OA the capacity to trigger coral and coralline bleaching, which has supported the widespread notion that OA is a stronger threat for these groups than thermal stress^38^,^39^. Our study does not support this interpretation for coralline algae, as we observed minimal impact of low pH on the photosynthetic descriptors (F_v_/F_m_, photosynthesis rates and algal pigmentation; Figs 4 and 5 and Tables S5, S6) under the moderate levels of light stress employed. Moreover, the direct impact of reduced pH on algal calcification was smaller than the impact caused by elevated temperature, in particular for the species most sensitive to light stress, which also showed the largest reductions in thallus photosynthesis (Fig. 5a).

The rhodolith *Neogoniolithon* sp. was the species that showed the most robust response to thermal and light stress, whereas the articulated alga *Amphiroa tribulus* and the CCA *Lithothamnion* sp. were particularly sensitive. In the physiological characterization performed before the experiment, the organisms of *Neogoniolithon* expressed a high-light condition, whereas *Lithothamnion* sp. presented physiological characteristics of a shade-adapted species, as reflected by the low irradiance levels required to induce similar excitation pressure in the photosynthetic membranes (PSII pressure *sensu*^40^ see Fig. 4). Samples of *Lithothamnion* sp. were collected on the side of or underneath coral colonies, under very low light conditions, whereas the rhodoliths of genus *Neogoniolithon* were growing in between the shallow and sparse seagrass meadow of the lagoon. Thus, the natural habitat of each species can explain their contrasting photosynthetic physiologies. In addition, the rapid changes in thallus illumination that rhodoliths suffer when they “roll” on the sediment implies that the species under study must have effective photoprotection mechanisms to respond to sudden exposures to high levels of solar radiation common in shallow reef habitats. Indeed, this capacity has been recently documented for a tropical rhodolith^41^. In contrast to the high-light condition, low light-adapted species have limited capacity to cope with light stress. This was confirmed for *Lithothamnion* sp. in this study, as we observed 100% thallus mortality when the experimental organisms were exposed to light conditions similar to those observed at 5–6 m depth (30–37% of surface irradiance, Es). Such strong mortality forced us to apply reduced levels of illumination (2% of Es) when we exposed the organisms of this species to the OA and thermal stress treatments. On the other hand, the high sensitivity showed by the articulated alga *Amphiroa tribulus* to light stress cannot respond to a similar shade-adapted physiology, as this species only required slightly lower irradiance levels than the rhodolith to maintain the optimal photosynthetic physiology of the control treatment (Fig. 4). This observation indicates that larger increases of light stress under elevated temperature or differences in the species ability to cope with light stress may explain the high accumulation of photodamage and the largest losses of algal photosynthesis and calcification found in this study for *Amphiroa tribulus* under elevated temperature (Fig. 5), and perhaps an important part of the large species-specific component documented for the response of coralline algae to thermal stress^4^,^11^,^31^,^32^.

In the physiological characterization of *A. tribulus,* we also observed that this species showed the largest increases in thallus respiration with temperature (Table 1, Fig. 3) and the largest impact of thermal-stress on the photosynthesis:respiration ratio (P_max_:R_L_; Fig. 5). Temperature enhances all metabolic rates through increasing enzyme activity and substrate availability (cf. Arrhenius equation^42^), and in the current analysis, we documented a significant variation among species and metabolic rates in response to temperature increases (Table 1), using the scaling factor known as Q_10_^43^ to characterize this effect. We also found the presence of species-specific physiological thresholds (Table 1, Fig. 3), as we observed, for example, that P_max_ was compromised in the three species at 32 °C (Fig. 3), but that respiration and calcification rates only declined in the CCA at the highest temperature (32 °C; Fig. 3). According to this, the physiological threshold of algal photosynthesis at 32 °C could explain the severe decline observed in the three species after exposing the organisms for 10 days to this temperature, along with the species-specific enhancement in photodamage. However, no adverse effect was detected at 32 °C for thallus respiration and calcification of the rhodolith and the articulated alga (Fig. 3). Thus, the reductions observed for these two species after 10 days at this temperature cannot be the result of a direct impact of temperature (Fig. 5), but to indirect responses associated with the severe reduction observed in algal photosynthesis. As the maintenance of a negative carbon balance in the algal thallus compromises the survival of the organism, a metabolic adjustment to resolve a suboptimal thallus condition (P_max_:R_L_ ≤ 1) may explain this response. In addition, extended periods of hypoxia within algal tissues may also limit aerobic respiration. Hence, the reduction observed in respiration may be an algal response to minimize the magnitude of carbon losses and/or the hypoxia impact under significant declines in photosynthesis production. This response would be equivalent at the tissue level to the self-thinning response of plant canopies^44^.

The origin of the variation observed in algal calcification is, however, less clear. We found calcification losses for the three coralline species and treatments, which were consistent with previous estimates^9^,^25^,^27^. Part of the changes observed may have resulted from the direct impact of the experimental treatments on algal photosynthesis, but other cellular processes not directly related to photosynthesis activity may have been also affected. Calcification is an energy-demanding process and photosynthesis provides the energy required to support the formation of carbonate skeletons. Algal respiration is an alternative source of energy to support coralline calcification, which requires the oxygen and substrates released by photosynthesis. Photosynthesis, thus, stimulates both calcification and respiration rates, but also can take advantage of the CO_2_ released by respiration. Similarly, calcification can enhance algal photosynthesis, if it acts as a carbon concentration mechanism, as proposed previously^45^. Accordingly, the three metabolic rates are coupled and need to vary in a coordinated manner in response to environmental changes. Linear associations between algal calcification and photosynthesis have been described in previous studies^46^,^47^. In this analysis we observed linear associations between calcification and algal respiration (Fig. 2c), but a non-linear coupling between algal calcification and photosynthesis (Fig. 2b). From this characterization, we also determined a species-specific photosynthetic threshold of algal calcification, termed the MPR (Table 1, Fig. 2b), below which decalcification activity was observed in the three species, as previously described for CCAs^18^. This MPR descriptor helped us to understand the differential impact caused by elevated temperature on the calcification rate of each species, as we observed that algal photosynthesis after 10 days under elevated temperature was: a) above the MPR value for the rhodolith *Neogoniolithon* sp.; b) close to its MPR value for *Lithothamnion* sp.; and c) below the MPR for *A. tribulus*, (Fig. 5, Table 2). Hence, the physiological characterization of the metabolic coupling between photosynthesis and calcification provides a quantitative model to describe the severity of the impact of thermal stress on coralline calcification, based on the estimation of its impact on algal photosynthesis. It also allows for the quantification of the magnitude of algal carbonate losses through the description of the variation in decalcification activity (Fig. 6). This model cannot yet account for the direct physiological effect of low pH on coralline calcification, and for any other potential negative effect of ocean acidification on the coupling mechanisms between the three metabolic rates. A deeper understanding of these mechanistic associations between algal photosynthesis, respiration and calcification will improve the interpretation of the experimental and natural observations.

With the current information presented in this study, we can conclude that the vulnerability of coralline algae to global change is primarily related to their high sensitivity to light stress under elevated temperature and the moderate adverse and direct effect of OA on algal calcification. This supports previous conclusions that OA significantly exacerbates the negative effects of global warming on marine organisms^6^. Considering the ecological significance of coralline algae for many coastal ecosystems, the impact of global change on coralline communities will affect a large variety of processes. Additional physiological characterizations for key species are still needed for the evaluation of the severity of the impact of global change on different coralline communities. These physiological descriptions will allow the development of a common physiological model to understand the response of coralline algae to global change. Species-specific responses are currently considered an important limitation for the understanding of the impact of global warming and OA on marine communities. This idea is leading to the conclusion that the only effective tools to identify general trends in this response are meta-analysis and long-term experiments. Although we recognize the utility of these approaches, we believe that only the understanding of the processes directly affected by each environmental threat will allow the integration of natural and experimental observations into a common explanatory model. Our study provides a first quantitative model for coralline algae, although still needs to incorporate the direct effect of reduced pH on algal calcification. Further research is necessary to elucidate the mechanism(s) and/or processes in coralline algae that are directly affected by lowered pH. However, our results do not support the attribution to OA^4^ as the trigger of coralline bleaching nor the interpretation that OA represents a greater threat to coralline algae than thermal stress^38^,^39^, but they highlight the extraordinary fragility of coralline communities in highly illuminated habitats such as coral reefs. The adverse effects reported here can have profound and negative consequences in these ecosystems, as they can compromise the contribution coralline algae to reef carbon budgets, reef cementation, coral recruitment and the maintenance of reef biodiversity.

## Materials and Methods

**Figure 1:**
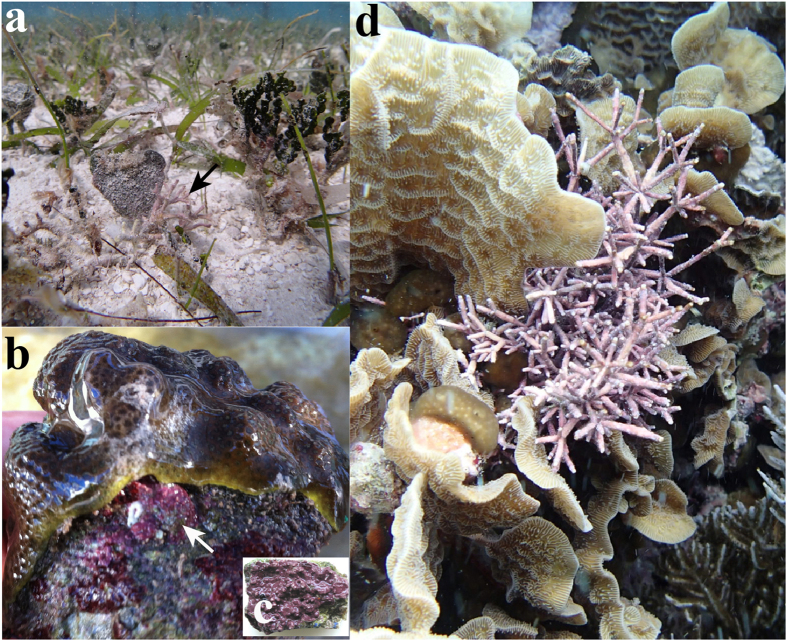
Images of the three coralline species (Rhodophyta) characterized in this study in their natural habitats. (**a**) Rhodolith (see arrow) belonging to the genus *Neogoniolithon* sp. collected at a depth of 3 m on sand sediments within sparse beds of the seagrass *Thalassia testudinum*. (**b**) The crustose coralline alga (CCA) belonging to the genus *Lithothamnion sp*. (see arrow) grows on the sides of/underneath *Orbicella faveolata/annularis* coral colonies at variable depths within the coral community but under very low light conditions. (**c**) Close-up of a CCA specimen used for the physiological characterization that was collected below an *Orbicella faveolata* skeleton. (**d**) The articulated alga *Amphiroa tribulus* growing within the branches of a colony of the coral *Agaricia agarites* at a depth of 5 m within the coral community.

Experimental organisms (Fig. 1) were collected in the reef lagoon of Puerto Morelos (Mexican Caribbean, 20°51’N, 86°55’W) in the summer 2013, when algae were already acclimated to the local monthly mean maximum (MMM = 30 °C)^35^. Rhodoliths belonging to *Neogoniolithon* sp. (Fig. 1a) were collected at 3 m depth by snorkeling. Samples of *Amphiroa tribulus* (articulated algae) and *Lithothamnion* sp. (crustose coralline, CCA, Fig. 1b–d) were collected by scuba diving at a depth of 5–6 m within the coral community of the back-reef. The articulated alga grew within *Agaricia spp.* Colonies while the CCA grew underneath or adjacent to coral colonies of *Orbicella faveolata* and *O. annularis*. After collection, the organisms were transported in black plastic bags to the outdoor tank facilities of the UNAM in Puerto Morelos. Prior to the experimental manipulation (August 2013), branches of rhodoliths and the articulated alga, and segments of the CCA of approximately 2–3 cm^2^, were cleaned of epiphytes and acclimated for seven days to the experimental control conditions. Changes in the maximum photosystem II (PSII) photochemical efficiency (F_v_/F_m_) were daily monitored at dusk with a submergible PAM fluorometer (Diving PAM Walz, Germany). The pre-acclimatory treatment was complete when F_v_/F_m_ reached a constant value, indicating successful thallus acclimation.

### Physiological determinations

Photosynthesis and respiration rates were determined polarographically in 20-ml water-jacketed chambers (DW3, Hansatech Instruments Ltd., Norfolk, UK) using Clark-type electrodes. The oxygen tension within the incubation chambers was maintained at 20–80% saturation by bubbling with N_2_ gas. A circulating bath with a controlled temperature system (RTE-100/RTE 101LP; Neslab Instruments Inc., Portsmouth, NH, USA) was used to maintain constant the incubation temperature (see Cayabyab and Enríquez^48^ for further details). Illumination was supplied with commercial 50-W halogen bulbs. A miniature PAR quantum sensor (Diving PAM, Walz, Germany) previously calibrated against an underwater light sensor (LI193-SAR, connected to a Data Logger LI-COR 1400, Lincoln, NE, USA) was used to calibrate the light within the chambers. Prior to the experimental analysis, we determined the photosynthetic response to irradiance (P vs. E curve) for each species (see Cayabyab and Enríquez^48^ for further details). This characterization enabled the selection of the light treatments and the irradiance required by each species for the determination of its maximum photosynthetic rate (P_max_). Accordingly, the maximum photosynthetic rate, P_max_, was determined during 45-min incubations at 600 μmol quanta m^−2^ s^−1^ for rhodoliths and the articulated algae and at 50 μmol quanta m^−2^ s^−1^ for the CCA. Post-illumination respiration (R_L_) was measured in darkness immediately after P_max_ determinations. Gross photosynthesis was calculated by adding R_L_ absolute values to net P_max_. Maximum calcification rates (G_max_) of algal thalli were determined using the alkalinity anomaly technique and a modified spectrophotometric procedure^49^ (see Enríquez and Schubert^50^ for further details). Coralline algae were incubated for 60 min at saturating irradiances in custom-made water-jacked acrylic chambers (30 ml) under a constant temperature. Filtered oceanic seawater (50 μm) collected the same day from the Yucatan current was used for these incubations. All metabolic rates were normalized to surface area, which was determined on a digital image using the software IMAGEJ^TM^ for rhodoliths and articulated algae. For the CCA, we used the aluminum foil technique.

### Analysis of the physiological coupling between metabolic rates

Simultaneous determinations of photosynthesis and calcification rates were performed at 30 °C under different irradiance levels (4 replicates per light condition and species). Each organism was sequentially exposed for 60-min to 5 increasing light intensities (4 subsaturating and one saturating light conditions for algal photosynthesis). Oxygen evolution was continuously recorded during the incubations. Seawater was collected at the beginning and end of each incubation, and stored for subsequent alkalinity analysis. After the last incubation (saturating light intensity), post-illumination respiration (R_L_) was also determined. For the description of the association between respiration and coralline calcification, the variation in thallus respiration was induced using different temperature of organisms previously exposed to saturation irradiance (Ek).

### Determination of the metabolic quotient (Q_10_) for photosynthesis, respiration and calcification

The analysis of the sensitivity of the three metabolic rates to temperature was determined examining their changes in P_max_, R_L_ and G_max_, at five different temperatures of incubation: 24 °C, 26 °C, 28 °C, 30 °C and 32 °C. This temperature range represents the seasonal fluctuation observed in the reef lagoon of Puerto Morelos (see description of the local temperature history for the period 1993–2005^35^). Each determination underwent a 30-min pre-incubation at each temperature right before the physiological incubation. Six replicates were used per temperature and species. Post-illumination respiration is a better descriptor of the effect of temperature on algal respiration as it is less affected by solute and oxygen limitation than dark respiration rates, as documented previously for symbiotic corals^51^.

### Experimental set-up: pH and thermal stress experiment

**Figure 2:**
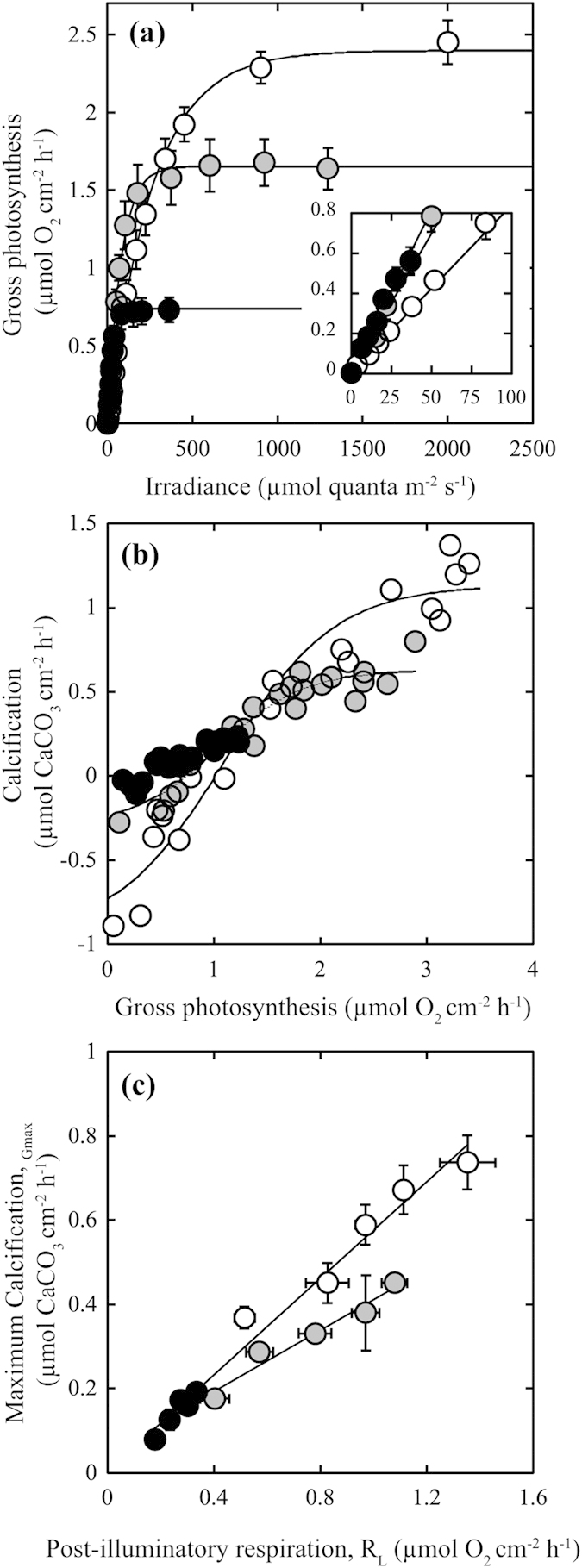
Description of the photosynthetic response to irradiance and the species-specific coupling among metabolic rates for each coralline growth form. (**a**) Photosynthetic responses to irradiance (P vs. E curve). The variation in photosynthetic efficiency (α, the slope of the linear increase at subsaturating light) is shown in the inserted graph. (**b**) Non-linear coupling between thallus calcification and gross photosynthesis. The trend lines represent sigmoidal fits (P < 0.05) with R^2^ = 0.93, 0.91 and 0.86 for rhodoliths, articulated alga and CCA, respectively. (**c**) Linear coupling between thallus calcification and post-illumination respiration. The trend lines represent linear regression fits (P < 0.05) for (i) pooled CCA and rhodolith data (R^2^ = 0.97) and (ii) data for the articulated alga *Amphiroa tribulus* (R^2^ = 0.97). Symbols represent mean values ± SEM (n = 4–8 determinations per metabolic rate): white circles indicate rhodoliths; grey circles indicate articulated algae; black circles indicate CCA.

**Figure 3:**
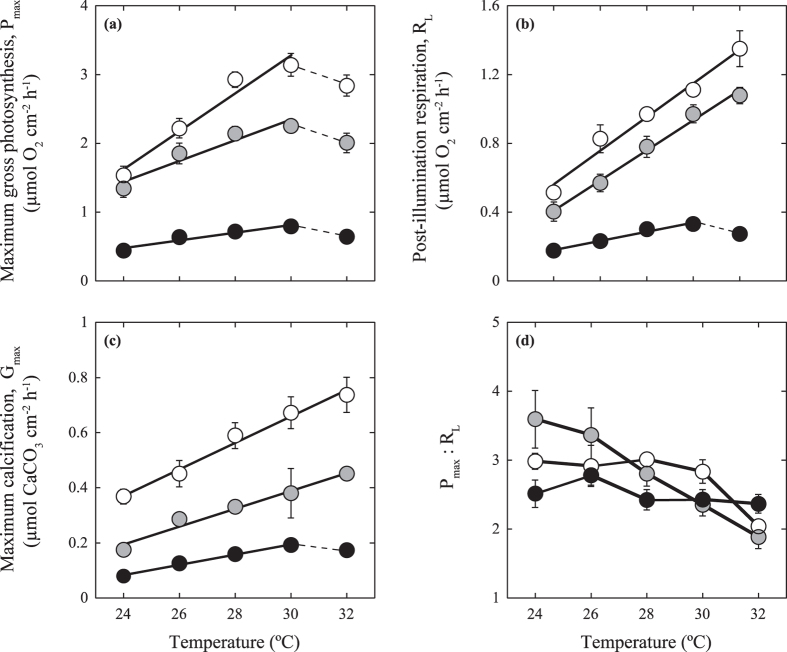
Scaling metabolic quotients (Q_10_) for photosynthesis, respiration and calcification rates. The symbols represent mean values ± SEM (*n* = 4–8) for (**a**) gross photosynthesis (P_max_), (**b**) post-illumination respiration (R_L_), (**c**) maximum calcification rates (G_max_), and (**d**) the photosynthesis to respiration ratio (P_max_:R_L_). The trend lines represent linear regression fits (see text for details). Dashed lines indicate metabolic rate decay. Symbols: white circles indicate rhodoliths, grey circles indicate articulated algae, and black circles indicate CCA.

**Figure 4:**
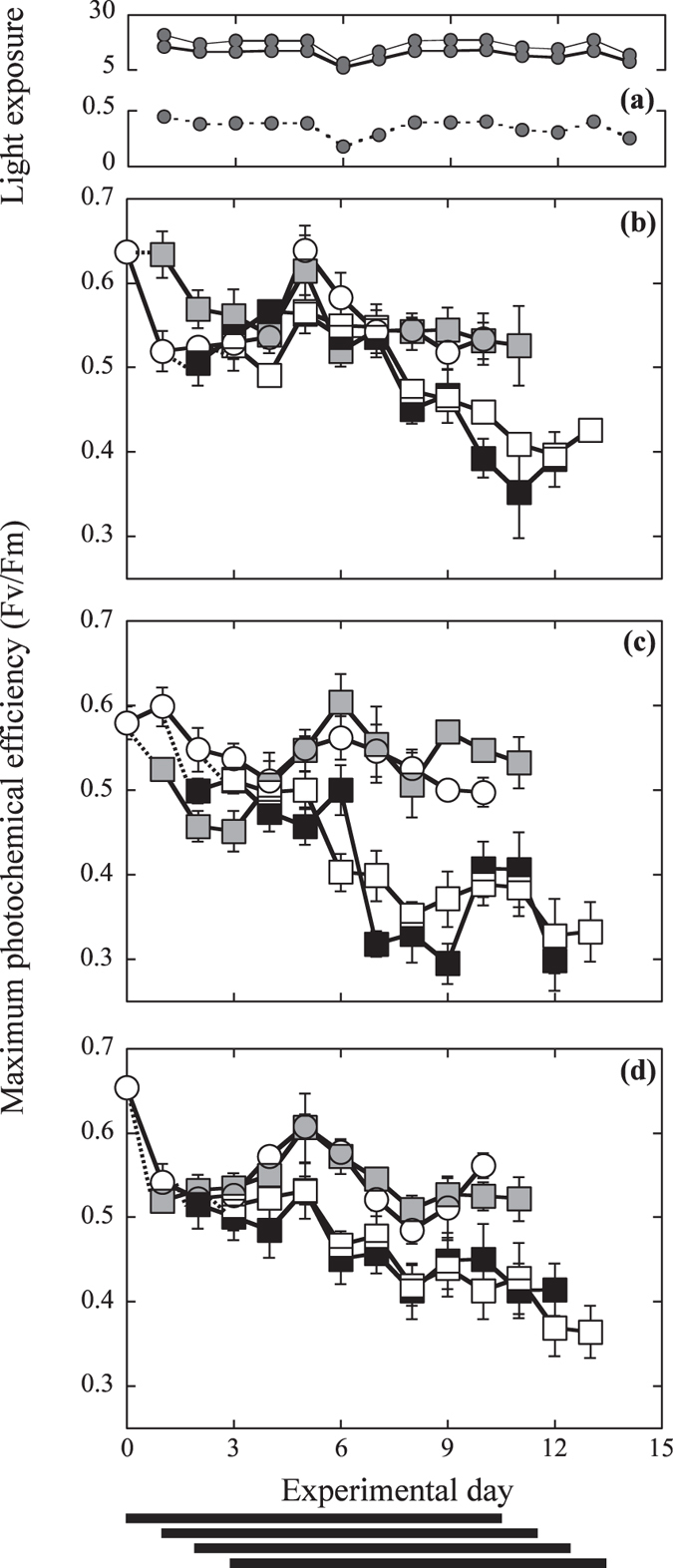
Variation in solar radiation and F_v_/F_m_ during the experiment. (**a**) The variation in daily light exposure (mol quanta m^−2^ d^−1^) and maximum photochemical efficiency of photosystem II, F_v_/F_m_, measured at dusk daily for rhodoliths (**b**), articulated alga (**c**) and CCA (**d**). Lines in (**a**) are grey for rhodoliths, black for the articulated alga, and dashed for CCA. Symbols in (**b–d**) are white circles for control treatments (ambient pH and 30 °C), grey squares for the low pH treatment (pH 7.9 and 30 °C), black squares for the thermal stress treatment (ambient pH and +2 °C [32 °C]), and white squares for the interaction between thermal stress and reduced pH (pH 7.9 and +2 °C [32 °C])). The dashed lines in (**b–d**) indicate F_v_/F_m_ values before the initiation of the corresponding treatment. The black horizontal bars at the bottom of the figure illustrate the beginning and end of each 10-day treatment. The symbols represent mean values ± SEM (*n* = 4–10 per experimental day).

**Figure 5:**
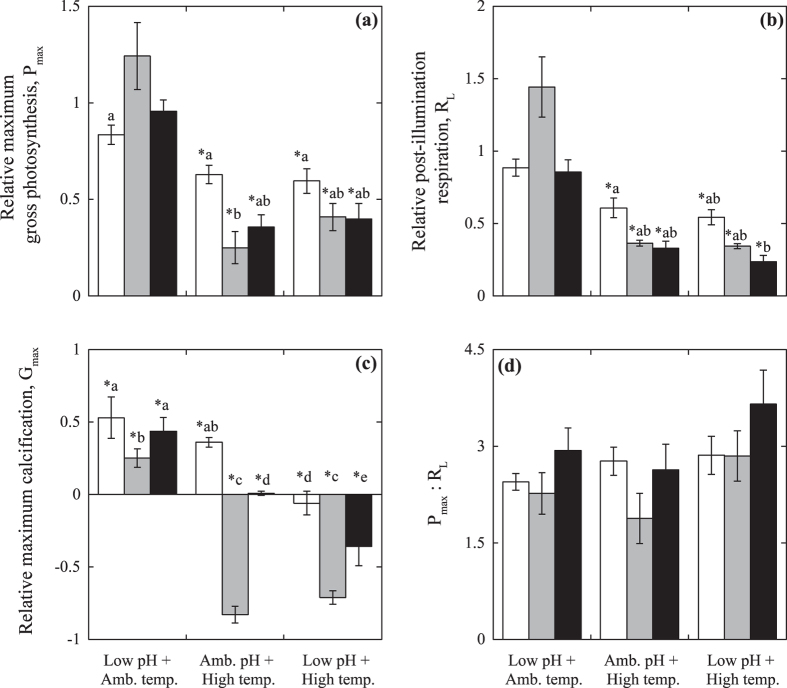
Coralline responses to experimental treatments. (**a**) Gross photosynthesis, P_max_; (**b**) post-illumination respiration, R_L_; (**c**) maximum calcification rate, G_max_; and (**d**) photosynthesis to respiration ratio, P_max_:R_L_. White bars indicate rhodoliths, grey bars indicate articulated alga, and black bars indicate CCA. Asterisks and letters denote significant differences relative to control organisms (Tukey HSD, P > 0.05). The bars in (**a–c**) represent the average ± SEM (*n* = 4–8 per treatment) relative change with respect to the control. The bars in (**d**) represent final absolute values. Negative values for the relative changes in algal calcification express the induction of decalcification activity quantified as increases in total alkalinity.

**Figure 6:**
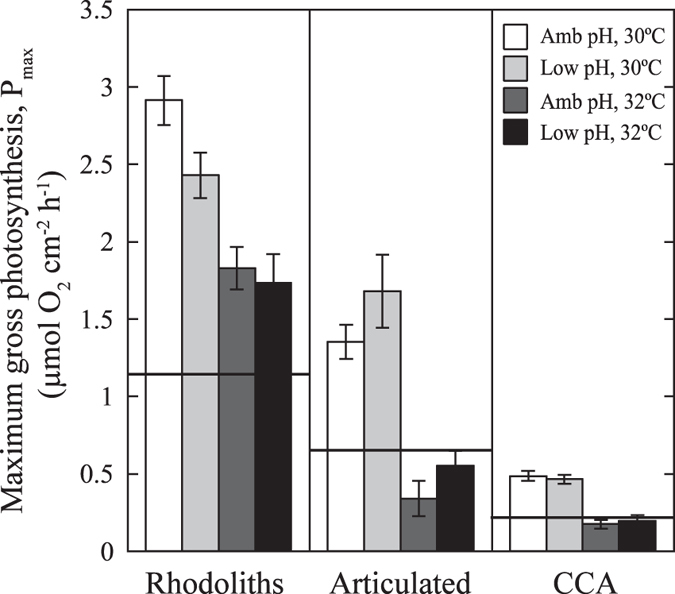
Changes induced in coralline photosynthesis rates relative to the estimated minimum photosynthesis requirement for coralline calcification (MPR). The bars represent mean values ± SEM of absolute photosynthesis after 10 days under the experimental treatments (n = 4–8). Horizontal lines represent the estimated *minimum photosynthesis requirement* (MPR) for each species from the sigmoidal fit (see Fig. 2b and text for details).

The pH and thermal stress experiments were performed at the UNAM mesocosm facilities of Puerto Morelos using 30-L tanks supplied with constant seawater from four 1000-L header tanks which had access to direct seawater flow from the reef lagoon. The average flow rate in the experimental tanks was 0.33 L s^−1^ with a turnover rate of about 90 s. After an initial pre-acclimation period, organisms (eight to ten organisms per species and tank; 16–20 per treatment) were exposed for 10 days to four treatments in a fully orthogonal two-factor design: 1) control treatment: 30 °C at sampling time (summer–August–average seawater temperature of the reef lagoon of Puerto Morelos^35^) and ambient pH (~8.1); 2) low pH treatment: control temperature (30 °C) and pH 7.9 (OA, B2 scenario according to IPCC 2007^52^); 3) thermal stress treatment: 32 °C (thermal anomaly of +2 above the local MMM) and ambient pH (~8.1); and 4) interaction treatment: 32 °C and pH 7.9. Two 30-L tanks were used per treatment. CCA and articulated algae were located in the same tanks, while rhodoliths were placed into independent tanks. We selected three experimental light treatments to induce similar and moderate light stress on each species (moderate PSII pressure *sensu*^40^) according to their particular photobiology, previously characterized in order to perform this experiment (P vs. E curve, see Fig. 2a). Three light attenuations of solar irradiance at the surface were achieved by means of neutral screens: 53% attenuance for the rhodolith (47% of surface irradiance, Es), 75% for the articulated alga (25% Es), and ~98% (2% Es) for the CCA. The very low light levels selected for the CCA reflect the extraordinary sensitivity showed by this species to light stress. Seawater pH and temperature were monitored every 10 min with a computer-based system connected to a wireless data acquisition card (National Instruments, USA) at a resolution of 0.1 °C units for temperature and 0.01 units for pH. Electronic valves (Sierra Instruments, Inc., Smart-Track 2) delivered bubbled CO_2_ to the reservoirs for pH control, and Thermo Scientific pH electrodes were calibrated at the beginning of the experiment using NBS buffers. Seawater temperature was controlled using commercial aquaria heaters (Process Technology, USA) located in the header tanks and connected to thermocouple sensors (J type, TEI Ingeniería, México). Superficial irradiance was recorded every 5 min with an underwater cosine-corrected sensor (LI193-SAR, LI-COR PAR) located in the tanks. The variation in diurnal solar radiation was expressed as changes in daily light exposure (mol quanta m^−2^ day^−1^). Due to the time required to complete the physiological determinations of each treatment, a 1-day delay was maintained before the initiation of the subsequent treatment. This delay allowed sufficient time for completion of the physiological characterizations of each treatment on the 10^th^ day after the initiation of each treatment. The following timeline was used, i) the control treatment started on day 0; ii) the pH treatment started on day 1; iii) the temperature treatment started on day 2, and iv) the interaction treatment started on day 3 (see Fig. 4). At the beginning of the experiment, samples were randomly distributed in the tanks. Every two days, the tanks were cleaned to prevent epiphyte accumulation, and water samples were collected for TA and pH determinations and carbonate chemistry analyses. F_v_/F_m_ was monitored daily at dusk. All measurements were taken 20–25 min after dusk (local time 20:00, <10 μmol quanta m^−2^ s-^1^) as this time of the day allows detection of the maximum value of the diurnal variation of the photochemical efficiency of PSII following similar methodologies used in previous studies^48^,^53^,^54^. At the beginning of the experiment and after 10 days under each experimental treatment, light-saturated gross photosynthesis (P_max_), post-illumination respiration (R_L_) and calcification (G_max_) rates were determined. Results are expressed relative to the control values (n = 6–8 replicates per treatment).

### Data analyses

Two-way ANOVA was used to analyze the effect of each experimental treatment on algal physiology. Tukey post-hoc tests enabled the identification of significant differences among treatments and/or species. Least-squares linear regression was used to describe the associations between metabolic rates and temperature, and ANCOVA analysis allowed the evaluation of differences among species. A sigmoidal model best described the association between algal photosynthesis and calcification^55^, and allowed estimation of the different parameters showed in Table 1. Q_10_ was calculated as the ratio between the values estimated at 34 °C and determined at 24 °C. Data were log-transformed to meet the assumptions of homoscedasticity and normality if necessary. Statistical calculations were performed using SPSS software (IBM Inc.), and a CO_2_ sys excel macro-sheet^56^ was used to calculate seawater carbonate chemistry.

## Additional Information

**How to cite this article**: Vásquez-Elizondo, R. M. and Enríquez, S. Coralline algal physiology is more adversely affected by elevated temperature than reduced pH. *Sci. Rep.* **5**, 19030; doi: 10.1038/srep19030 (2015).

## Supplementary Material

Supplementary Information

## Figures and Tables

**Table 1:** Metabolic quotients (Q_10_) and parameters derived from the sigmoidal fit describing the species-specific metabolic coupling between calcification and photosynthesis rates (see Fig. 2b) for: *Neogoniolithon sp.* (rhodolith), *Amphiroa tribulus* (articulated) and the CCA *Lithothamnion sp*.

*Metabolic quotient (Q*_*10*_)	Rhodoliths	Articulated	CCA
Photosynthesis (P_max_)	2.53	2.29	2.06
Post-illumination Respiration (R_L_)	2.57	2.91	2.31
Calcification (G_max_)	2.16	2.51	2.97
*Photosynthesis-Calcification coupling*			
Maximum calcification rate (G_max_, μmol CaCO_3_ cm^−2^ h^−1^)	1.21 ± 0.12	0.62 ± 0.04	0.27 ± 0.06
Minimum photosynthesis requirements, MPR^1^ (μmol O_2_ cm^−2^ h^−1^)	1.15 ± 0.16	0.63 ± 0.07	0.23 ± 0.08
Centroid (μmol O_2_ cm^−2^ h^−1^)	1.13 ± 0.13	1.04 ± 0.08	0.47 ± 0.11
* Light enhanced calcification* (r_G:P_) Rate of increment in calcification as a function of photosynthesis activity (μmol CaCO_3_ (μmol O_2_)^−1^)	0.46 ± 0.07	0.38 ± 0.05	0.27 ± 0.05

^1^Minimum photosynthesis requirements refers to the x value of photosynthesis for y = 0 (no calcification or decalcification activity) of the linear regression between calcification and photosynthesis.

**Table 2:** Mean ± SE (n = 4–8 replicates) of the absolute values of coralline metabolic rates for *Neogoniolithon sp.* (rhodolith), *Amphiroa tribulus* (articulated) and the CCA *Lithothamnion sp*, after being exposed for 10 days in semi-controlled conditions to reduced pH (=7.9) and + 2 °C of seawater temperature above MMM = 30 °C (thermal-stress).

*Metabolic rate*		Ambient pH, 30 °C	Low pH, 30 °C	Ambient pH, 32 °C	Low pH, 32 °C
Post-illumination respiration R_L_ (μmol O_2_ cm^−2^ h^−1^)					
	Rhodoliths	1.13 ± 0.07	1.003 ± 0.06	0.68 ± 0.07	0.61 ± 0.05
	Articulated	0.55 ± 0.07	0.79 ± 0.11	0.20 ± 0.01	0.18 ± 0.008
	CCA	0.19 ± 0.03	0.16 ± 0.01	0.06 ± 0.009	0.04 ± 0.008
Maximum gross photosynthesis, P_max_ (μmol O^2^ cm^−2^ h^−1^)					
	Rhodoliths	2.91 ± 0.15	2.42 ± 0.14	1.82 ± 0.13	1.73 ± 0.18
	Articulated	1.35 ± 0.10	1.67 ± 0.23	0.33 ± 0.11	0.55 ± 0.09
	CCA	0.48 ± 0.03	0.46 ± 0.02	0.17 ± 0.03	0.19 ± 0.03
Maximum calcification rate, G_max_ (μmol CaCO_3_ cm^−2^ h^−1^)					
	Rhodoliths	0.57 ± 0.01	0.30 ± 0.08	0.20 ± 0.01	−0.03 ± 0.04
	Articulated	0.30 ± 0.02	0.07 ± 0.01	−0.25 ± 0.01	−0.24 ± 0.01
	CCA	0.14 ± 0.06	0.07 ± 0.01	0.01 ± 0.01	−0.05 ± 0.01

**Table 3:** Two-way ANOVA tests analysing direct and combined effects of low pH and thermal-stress (+2 °C) on three coralline species: *Neogoniolithon sp.* (rhodolith), *Amphiroa tribulus* (articulated) and the CCA *Lithothamnion sp*. P_max_, maximum gross photosynthesis (μmol O_2_ cm^−2^ h^−1^); R_L_, Post-illumination Respiration (μmol O_2_ cm^−2^ h^−1^); and G_max_, maximum calcification rate (μmol CaCO_3_ cm^−2^ h^−1^).

	Rodoliths		F	*P*	Articulated		F	*P*	CCA	MS	F	*P*
	df	MS			df	MS			df			
Source of variation												
P_max_												
pH	1	0.564	3.35	0.08	1	0.255	1.75	0.199	1	<0.001	0.001	0.981
Temp.	1	5.319	31.5	<0.05*	1	6.298	43.44	<0.05*	1	0.603	83.014	<0.05*
pH*temp.	1	0.254	1.5	0.232	1	0.001	0.009	0.926	25	0.003	0.428	0.519
Error	24	0.168			21	0.145				0.007		
R_L_												
pH	1	0.07	1.804	0.192	1	0.012	0.677	0.419	1	0.005	1.522	0.229
Temp.	1	1.173	30.393	<0.05*	1	1.478	84.274	<0.05*	1	0.112	36.563	<0.05*
pH*temp.	1	0.005	0.132	0.72	1	0.028	1.619	0.216	1	<0.001	0.023	0.882
Error	24	0.039			21	0.018			25	0.003		
G_max_												
pH	1	0.308	19.17	<0.05*	1	0.074	29.6	<0.05*	1	0.029	18.235	<0.05*
Temp.	1	0.59	36.62	<0.05*	1	1.207	483.14	<0.05*	1	0.111	69.577	<0.05*
pH*temp.	1	0.001	0.056	0.816	1	0.089	35.58	<0.05*	1	<0.001	0.074	0.788
Error	16	0.016			22	0.002			23	0.002		
