# Supplementary material for: Coralline algal physiology is more adversely affected by elevated temperature than reduced pH
Source: Sci Rep. 2016 Jan 7;6:19030. doi: 10.1038/srep19030 (PMC4704045; doi:10.1038/srep19030)
Supplement: Supplementary Information [file srep19030-s1.pdf]

Supporting information: Román Manuel Vásquez-Elizondo and Susana Enríquez

Coralline algal physiology is more adversely affected by elevated temperature than reduced pH

Methods

Physiological determinations:

Photosynthesis-irradiance curves (P vs E) were determined following the methodology described by Enríquez *et al.* (2002) and Cayabyab and Enríquez (2007). Four to six replicates (organisms) were used for the determination of each experimental curve. Each sample was exposed sequentially to 14-15 light treatments for 10-15 min, from dark conditions (dark respiration R_D, 15 min.) to a progressive increase in the irradiance levels. Oxygen evolution was monitored continuously and the slope of the changes observed was used to calculate the rate of oxygen evolution or consumption at each treatment. After exposing the samples to the different irradiances, oxygen evolution was measured again in the dark to determine the post-illumination respiration (R_L). The maximum photosynthetic rate (P_{max}) was calculated from the average maximum values above saturating irradiance E_k (n=3-4). The linear slope of the curve (α) was estimated using a least-squares regression analysis, for the values under subsaturating irradiance that showed a linear increase with irradiance. Finally, saturation and compensation irradiances were calculated as the ratio of P_{max}: α and R_D:α. Data is presented as gross photosynthesis subtracting the R_D to the net photosynthesis values.

Figure S1:

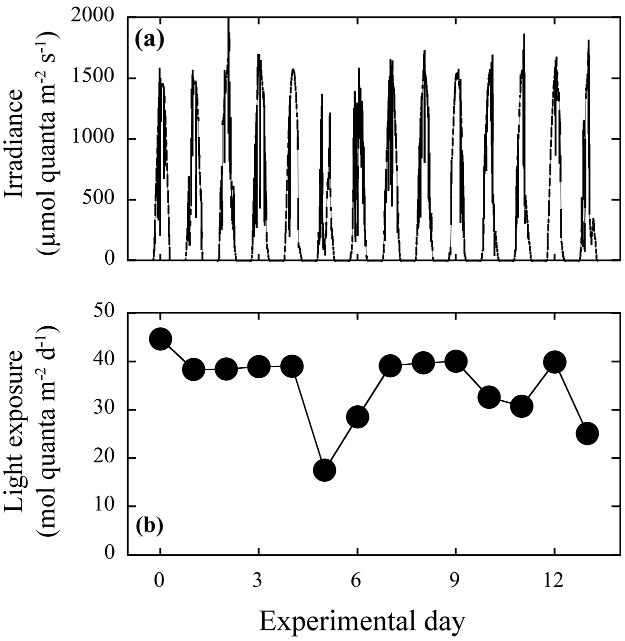

Figure S1. Variation in surface solar radiation during the experiment. Variation in diurnal surface irradiance (a) and variation in daily light exposure at the surface (b). Data from the Oceanographic and Meteorological Academic Service (SAMMO) of the UASA-UNAM.

Figure S2:

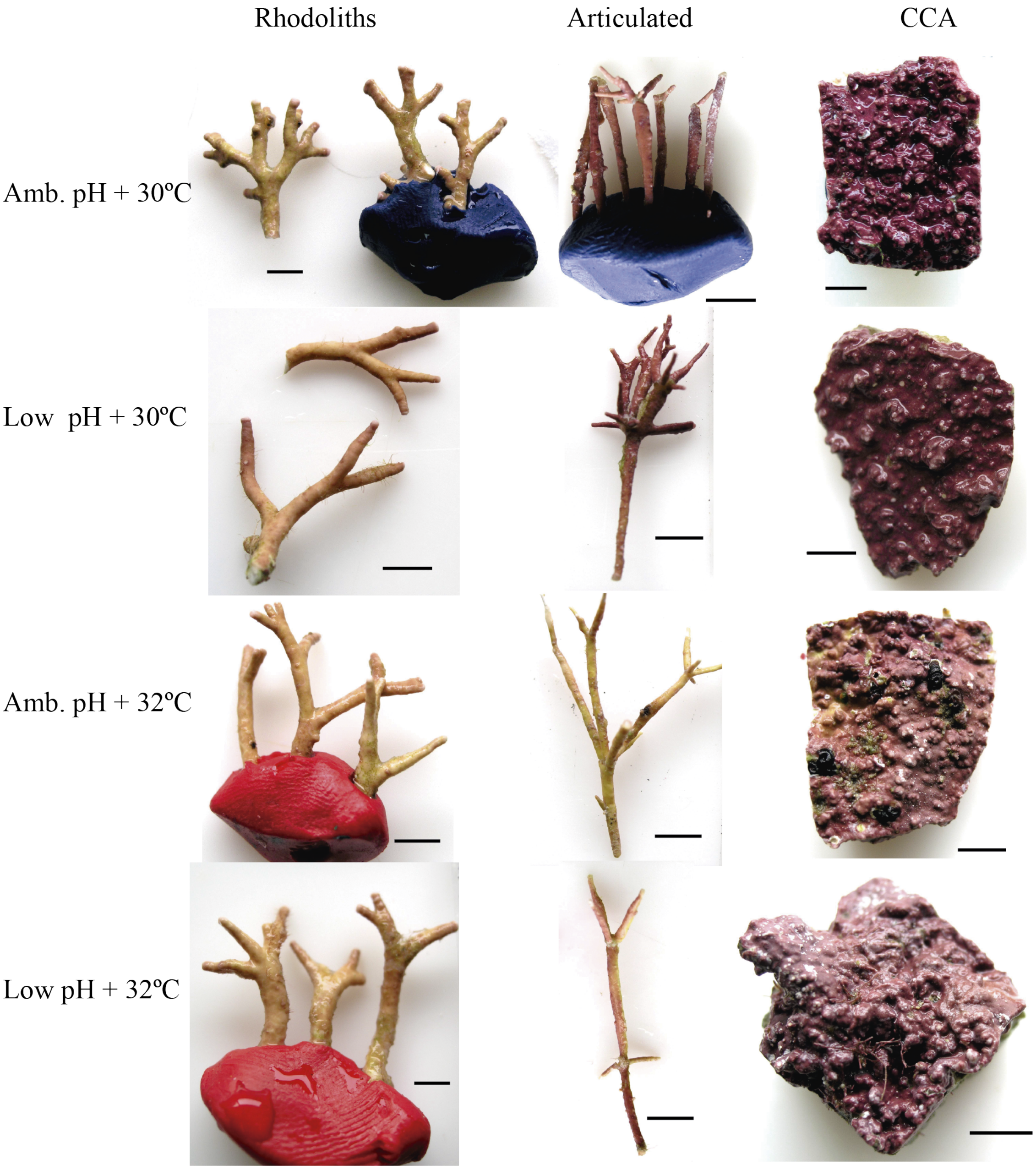

Figure S2. Changes in thallus coloration of the experimental organisms exposed to low pH and thermal stress. Control temperature = 30ºC, Amb pH = ~ 8.1; Thermal stress = 32ºC and low pH = 7.9.

Table S1: Physiological parameters derived from the photosynthesis vs irradiance curve (P vs E curve). Different letters indicate statistical differences among coralline morphotypes (One-way ANOVA, P<0.05, Tukey Post-hoc P<0.05).

	Rhodoliths	Articulated	CCA
P_{max} (µmol O_2 cm^{-2} h^{-1})	2.32 ± 0.11[a]	1.73 ± 0.16[b]	0.75 ± 0.008[c]
Alpha (µmol O_2 cm^{-2} h^{-1}) (µmol quanta m^{-2} s^{-1})$^{-1}$	0.009 ± 0.0003[a]	0.015 ± 0.0001[b]	0.016 ± 0.0001[b]
E_k	255 ± 9.3[a]	118 ± 15.9[b]	46.3 ± 1.9[c]
R_L (µmol O_2 cm^{-2} h^{-1})	0.76 ± 0.12[a]	0.77 ± 0.10[a]	0.43 ± 0.06[b]

Table S2. Least-square regression analyses for the description of the Q_{10} factor for each metabolic rate and coralline species (short-term incubations). Significant differences between slopes are indicated with different letters.

Metabolic rate	Morphotype	Slope	SE	R^2	*P*	n
Post illumination respiration (R_L)						
	Rhodoliths	0.098 ± 0.010[a]	0.157	0.76	<0.001	30
	Articulated	0.088 ± 0.008[a]	0.126	0.79	<0.001	30
	CCA	0.027 ± 0.004[b]	0.039	0.69	<0.001	24
Maximum calcification rate (G_{max})						
	Rhodoliths	0.040 ± 0.008[a]	0.127	0.46	<0.001	27
	Articulated	0.033 ± 0.007[b]	0.092	0.49	<0.001	23
	CCA	0.018 ± 0.003[c]	0.033	0.60	<0.001	21
Gross photosynthesis (P_{max})						
	Rhodoliths	0.276 ± 0.032[a]	0.346	0.77	<0.001	23
	Articulated	0.150 ± 0.058[b]	0.303	0.55	<0.001	24
	CCA	0.057 ± 0.004[c]	0.079	0.72	<0.001	24
Photosynthesis respiration ratio (P:R)						
	Rhodoliths	-0.098 ± 0.028	0.434	0.77	0.002	29
	Articulated	-0.221 ± 0.044	0.303	0.67	0.001	30
	CCA	-	-	-	0.203	30

Table S3. ANCOVA analyses testing the differences among species in the colinearity observed between different metabolic rates.

Metabolic rate		*df*	MS	F	*P*
P_{max}	Rhodoliths vs Articulated	*1, 43*	0.949	8.85	**0.05**
	Rhodoliths vs CCA	*1, 43*	2.88	46.51	**0.001**
	Articulated vs CCA	*1, 44*	2.88	10.68	**0.002**
R_L	Rhodoliths vs Articulated	*1, 56*	0.015	0.727	0.397
	Rhodoliths vs CCA	*1, 50*	0.456	26.71	**<0.05**
	Articulated vs CCA	*1, 50*	0.456	30.73	**<0.05**
G_{max}	Rhodoliths vs Articulated	*1, 43*	0.949	8.95	**<0.05**
	Rhodoliths vs CCA	*1, 43*	2.88	46.55	**<0.05**
	Articulated vs CCA	*1, 44*	0.527	10.68	**<0.05**

Table S4. Carbonate chemistry for the different experimental treatments. For temperature and pH n=14 (daily averages). Carbonate system parameters n=8 (± SE).

Treatment	Temperature (ºC)	pH_{NBS}	Total alkalinity (µmol kg^{-1})	PCO_2 (µatm)	TCO_2 (µmol kg^{-1})	Ω_{cal}	CO_3 (µmol kg^{-1})
Ambient pH, 30ºC (Control)	29.98 ± 0.07	8.12 ± 0.004	2333.3 ± 8.9	488.1 ± 7.1	2029.2 ± 9.7	5.32 ± 0.03	218.8 ± 1.4
Low pH, 30ºC	30 ± 0.04	7.89 ± 0.001	2315 ± 13.1	902.4 ± 7.8	2133.3 ± 13.1	3.45 ± 0.01	141.9 ± 0.6
Ambient pH, 32ºC	31.95 ± 0.06	8.18 ± 0.01	2317 ± 17.8	415.23 ± 16.9	1962.8 ± 23	6.12 ± 0.07	250.9 ± 3
Low pH, 32ºC	32 ± 0.03	7.9 ± 0.0004	2304.5 ± 17.8	895 ± 7.1	2109.2 ± 16.8	3.66 ± 0.02	150.1 ± 1.2

Table S5. Variation in the photosynthetic pigments of the experimental organisms exposed for 10 days to different experimental treatments. Chlorophyll *a* and Antennae pigments such as Phycoerythrin + Phycocyanin + Allophycocyanin, are expressed in mg pigment m^{-2}. Data describe the average ±SEM of n=6-8 replicates. ND denote the lack of data, as we lost during the preservation the experimental CCA organisms exposed to low pH + 30ºC. Different letters indicate significant differences among treatments (One way ANOVA, Tukey HSD, P<0.05)

		Amb pH + 30ºC	Low pH + 30ºC	Amb pH + 32ºC	Low pH + 32ºC
	Chl a				
Rhodoliths		20.8± 2.2	19.3± 1.6	15.5± 1.7	15.1± 1.6
Articulated		72.9± 5.3^{ab}	76± 5.3^{a}	31.6± 3.6^{c}	\55± 2.1^{b}
CCA		88.6± 9.4^{a}	ND	55.6± 4^{b}	63.1± 10.6^{b}
	Antennae				
Rhodoliths		81.9± 5.8	81.5± 6.7	67.2± 10.9	73.5± 5.1
Articulated		492.6± 36.7^{a}	495.9± 22.1^{a}	319.3± 30.5^{b}	394.6± 19.4^{a}
CCA		674.1± 92.9^{a}	ND	348.8± 46.3^{b}	432.9± 60.7^{a}

Table S6. Two-way ANOVA tests analyzing direct and combined effects of low pH and thermal-stress (+2ºC) on maximum photochemical efficiency (F_v/F_m) at the end of the experiment on the three coralline species: *Neogoniolithon sp.* (rhodolith), *Amphiroa tribulus* (articulated) and the CCA *Lithothamnion sp*.

	Rhodoliths				Articulated				CCA			
Source of Variation	df	MS	F	*P*	df	MS	F	*P*	df	MS	F	*P*
Temp	1	0.082	12.4	**0.002**	1	0.24	73.42	**<0.01**	1	0.14	47.3	**<0.01**
pH	1	0.001	0.15	0.695	1	0.007	2.175	0.154	1	0.012	4.16	0.054
pH*temp.	1	0.003	0.38	0.541	1	$1.5*10^{-6}$	<0.001	0.983	1	<0.001	0.05	0.811
Error	20	0.131			26	0.07			25	0.065		

Table S7. T-test analyses for the comparison of the control organisms (ambient pH + 30ºC, day 10) between initial (day 0) and final experimental day.

Metabolic rate		df	*t*	*P*
P_{max}	Rhodoliths	13	0.052	0.959
	Articulated	13	1.65	0.123
	CCA	14	-0.74	0.46
R_L	Rhodoliths	13	-0.98	0.36
	Articulated	13	1.5	0.148
	CCA	14	-0.6	0.556
G_{max}	Rhodoliths	10	0.447	0.665
	Articulated	9	0.821	0.433
	CCA	10	0.774	0.457

Table S8. Two-way ANOVA test for the analysis of the tank effect of this experimental approach, for the different metabolic rates and coralline algal species analysed.

		df	SM	F	*P*
P_{max}	Rhodoliths	*4,20*	*0.19*	1.11	0.359
	Articulated	*4,18*	0.15	0.003	0.954
	CCA	*4,21*	0.01	1.39	0.27
R_L	Rhodoliths	*4,20*	0.048	1.3	0.301
	Articulated	*4, 18*	0.026	0.73	0.579
	CCA	*4,21*	0.003	1.18	0.346
G_{max}	Rhodoliths	*4,12*	0.023	0.017	0.899
	Articulated	*4, 17*	0.004	2.18	0.115
	CCA	*4,19*	0.002	1.15	0.363
